# Increase in Preterm Birth during Demographic Transition in Chile from 1991 to 2012

**DOI:** 10.1155/2015/845968

**Published:** 2015-08-27

**Authors:** Paulina López Orellana

**Affiliations:** Escuela de Obstetricia y Puericultura, Universidad de Valparaíso, Valparaíso, Chile

## Abstract

*Introduction*. Universally mothers at 35 years or more have had higher maternal and perinatal risks. This study analyzed the trend of this group in maternal population and determined their risk of having premature children, during the demographic transition period in Chile. *Materials and Methods*. Epidemiological study conducted in the population of simple live births registered in the Chilean National Database Births of 1991–2012. Analyses were performed in three categories of maternal age: 35 or more, under 35, and 20 to 29 years. The risk of prematurity was measured by crude and Adjusted Odds Ratio from logistic regression model. *Results*. Mothers aged 35 and older increased in population from 10.6% in 1991 to 16.7% in 2012 and presented an overall prevalence of preterm delivery of 6.7%, higher prevalence than 20–29 age group (4.7%). In aging mothers, the Odds Ratio for preterm birth adjusted for education, marital status, and parity was 1.68 (95% CI (1.66–1.70)) compared to mothers aged 20–29. All differences were significant (*p* < 0.001). *Conclusions*. During Chilean demographic transition, mothers aged 35 or older increased steadily and significantly maintaining higher risks of preterm births. Policies to prevent and monitor the late motherhood could contribute to stopping the current trend.

## 1. Introduction

In most industrialized countries, the characteristics of the maternal population changed in the last three decades and this had a broad impact on maternal and child health [1–3].

In Chile, the demographic and health statistics indicated changes that were expressed in a decrease in the birth rate and fertility, the latest age at first birth, and other modifications of socioeconomic order such as increased schooling and the female employment rate. These changes resulted from the demographic and epidemiological transition that was occurring since the 80s; in this context, increasing of maternal age corresponded to the demographic structure of population aging [4, 5].

Risks associated with increased late motherhood (from 35 years on) resulted in increased maternal morbidity and mortality. In Chile, advanced maternal age was becoming more difficult to decline in maternal mortality since 2003 where the lowest rate of 12.7/100,000 live births was registered. This was stated by various publications by E. Donoso which showed that the commitment Chile acquired by assuming the 5th Millennium Development Goal by reducing maternal mortality to 75% in 2015 (reaching the rate of 9.9/100,000 births alive) has been impossible to fulfill because of age 4–6 factor and, besides, mortality was concentrated in this group of mothers. Therefore, 51% of maternal deaths that occurred between 2000 and 2009 were associated with comorbidities that mostly affect mothers over 35 years [6–8].

Along with maternal risk is the high risk of perinatal and neonatal death more specifically [9, 10] with prematurity as first direct or indirect cause. In Chile, the participation of prematurity in perinatal and neonatal mortality increased from 65% in 1999 to 83% in 2004 [11].

The risk was due to the increase in this group of mothers of preterm birth (less than 37 weeks of gestational age), low birth weight (less than 2500 g) with intrauterine growth restriction (fetal weight estimated gestational age below the 10th percentile), and congenital malformations [11, 12].

Since premature birth rarely responded to a single cause, it considered the presence of other related factors to age, such as chronic diseases, overweight, parity, and socioeconomic status, among others [13, 14].

In Chile, few studies have centered on the impact of advanced maternal age on the births. A previous publication in the same maternal population (1991 to 2008) showed the highest risk for mothers over 38 of having very preterm child (28–31 weeks gestational age) and child with low birth weight [15].

## 2. Objective

The main objective was to analyze the trend of maternal age population that held newborns unique and alive new born during 1991 to 2011 to determine the risk of having a premature infant with birth among mothers aged 35 or more by comparison to mothers aged 20–29.

## 3. Methods

Epidemiological study of analytic and transversal cut, was made in the population of live births registered in the National Database of Live Births in Chile, during the period 1991–2012. Multiple births and births that do not correspond to the definition of live birth, under 22 weeks gestational age and/or with less than 500 g [16] were excluded.

The main explanatory variable is the maternal age which was analyzed in three categories: 35 or more, under 35, and 20 to 29 years.

The main result was preterm birth defined by World Health Organization, as birth less than 37 weeks gestational age.

First, the distribution of three categories of maternal age annually was described and the evolution of prematurity was further evaluated by group of mothers. The trend of prematurity was evaluated with the Prais-Winsten Test. The differences in rates of preterm between 1991 and 2012 were measured by the relative change (% change).

The risk of preterm delivery of maternal population aged 35 or older was determined by the crude and Adjusted Odds Ratio (OR) extracted from a model of logistic regression. The adjustment variables were parity, marital status, and educational level of the mother. The reference group was mothers aged 20–29 years, considered as a group of low obstetric risk.

## 4. Results

Out of a total of 5,459,001 live births in the study period, 95,821 were excluded and 5,363,180 single live births were analyzed.

**Figure 1:**
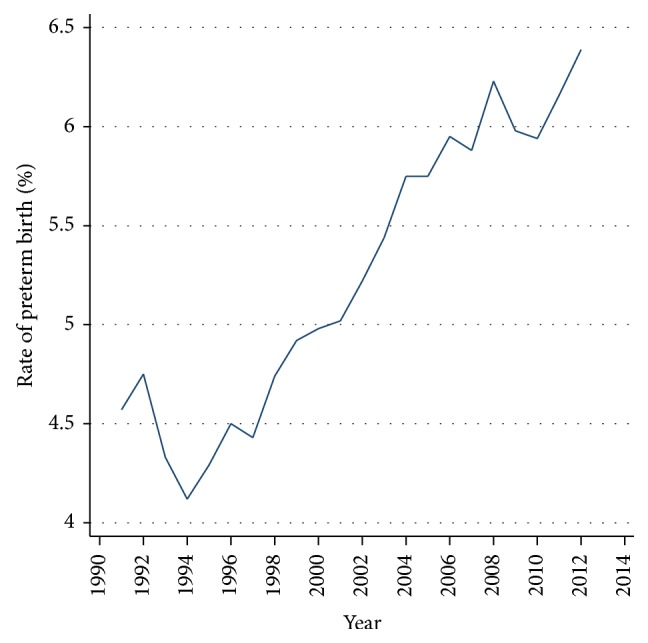
Global rate (%) of preterm birth per year. Chile, single live births 1991–2012. Prais Winsten Test with *p* < 0.001.

Mothers of 35 or more increased from 10.6% in 1991 to 16.6% in 2012 (Table 1). Similarly, mothers aged 20–29 years, considered as a group of low-risk obstetrics, decreased significantly (Table 1). The global rate (%) of preterm birth increased steadily and significantly from 4.7% to 6.4% (Figure 1).

**Figure 2:**
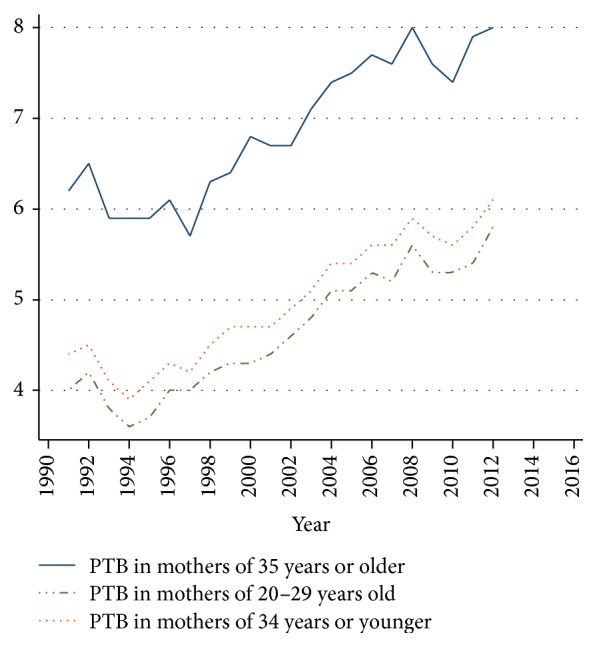
Rates of preterm birth in three groups of maternal age. Chile, single live births 1991–2012.

Figure 2 shows that late mothers held higher global rates of preterm birth regarding mothers under 35 years. All differences were significant (*p* < 0.001). Although it was observed that prematurity significantly increased over time in all groups studied, the group over 34 had a higher initial rate with 6.2% in 1991 and reached 8.0% in 2012 which corresponded to an increase of 29%. This trend was evolving, so it was estimated that prematurity in this group could reach 8.5% in 2015, since the estimated global increase per year was 0.2% by logit function (*p* < 0.000).

In terms of risk measurement, compared to mothers aged 20–29 years, the Adjusted Odds Ratio (AOR) for parity, marital status, and education level, for preterm birth in women aged 35 years and over, was 1.68 (95% CI (1.66 to 1.70)).

**Figure 3:**
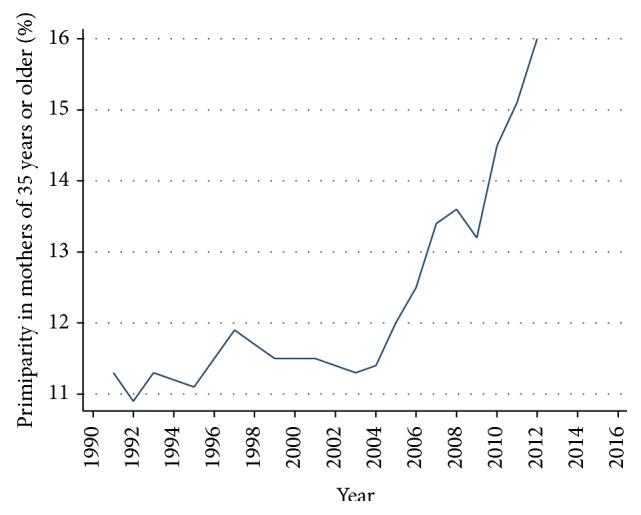
Increased late primiparity in mothers of 35 and older. Chile, single live births 1991–2012. Prais-Winsten Test with *p* = 0.005.

The risk increases with age and primiparity and it was observed that there was a significant increase in primiparity in mothers aged 35 and over, from 11.3% to 16.0% at the end of the observed period. In this specific group of primiparae, the risk of having a premature child increased significantly: AOR = 2.10 (95% CI (1.93 to 2.27)). This trend became more evident since 2009 (Figure 3).

In summary, in Chile, over the past two decades, premature births increased overall and in all ages. The population groups of mothers of 35 or older and also primiparas increased and this group had higher rates of preterm delivery over time.

The adjusted OR for level of education and marital status remained unchanged, which meant that elderly maternal age is a premature birth risk factor independent of schooling and marital status but it was not independent from parity, because primiparity significantly increased the risk of mothers of 35 years.

## 5. Discussion

The present study provided information to evaluate emerging problem of maternal and perinatal health, a public health problem as part of changes in population in demographic transition period [17]. Indeed, Chile was completing the demographic transition and countries that have already done so, like Norway, reached one-third of maternal population older than 34, close to ratios which were observed in Central Europe.

The increase in mothers of 35 or more deserved special attention, mainly in countries with limited resources or those at low income, since the new situation leads to multiple challenges to obstetric and perinatal care such as increasing human teams and highly specialized technicians for both the mother and the newborn. It was therefore necessary to focus prevention strategies towards women of childbearing age, as appropriate and relevant information about maternal and perinatal risk associated to age, older age at first birth.

Large databases allow seeing the demographic reality, a reality on a large scale, and therefore constitute strong evidence. However, they have a limitation which is not allowing access to more specific risk profiles, especially biological ones. Otherwise, it has been known by national and international publications that hypertensive and metabolic pregnancy disorders, especially diabetes mellitus and obesity, are the main risk factors added to advanced maternal age [18–21].

Studies from industrialized countries indicated not only these diseases as the most prevalent and described similar results but also risks associated with treatments for hypofertility used in elderly women that led to a significant increase in multiple pregnancies.

Moreover, in developed countries, increase of maternal age and late primiparity are related to the more important demand of assisted reproductive technologies (ARTs). In Chile, medical offer of assisted procreation has increased since the decade of 90s in major cities. This offer remains with a high individual cost; however, it has a growing acceptance by the population [22].

Therefore, the contribution of this group to national situation of preterm birth in the recent twenty years seems to be relatively poor. Indeed, those births have not reached an average of 500 per year and 26% of them were born prematurely [23]. These preterm births were specifically associated with multiple births. In this study, this group of births has been excluded.

The results presented could be useful in other Latin American countries that shared similar realities with Chile and started the process of demographic transition in the case of Bolivia, Ecuador, Peru, and Venezuela just to mention a few [24].

Future researches should investigate the factors influencing the moment of motherhood in the population, usually noncollected information, in order to target prevention efforts.

## Figures and Tables

**Table 1:** Evolution of single live births and maternal age by year. Chile, 1991–2012.

Birth year	Number of births	% 20–29	% < 35	% ≥ 35
1991	272574	**56.9**	89.4	**10.6**
1992	267356	**55.8**	89.0	**11.0**
1993	264541	**54.9**	88.6	**11.4**
1994	262969	**54.3**	88.5	**11.5**
1995	255581	**53.1**	87.9	**12.1**
1996	254635	**52.2**	87.4	**12.6**
1997	250274	**51.1**	86.9	**13.1**
1998	247509	**50.2**	86.6	**13.4**
1999	241036	**49.5**	86.0	**14.0**
2000	239370	**48.8**	85.4	**14.6**
2001	236807	**48.0**	84.8	**15.2**
2002	229835	**48.0**	84.5	**15.5**
2003	225555	**47.5**	84.0	**16.0**
2004	222297	**46.9**	83.9	**16.1**
2005	223173	**46.7**	84.0	**16.0**
2006	223213	**46.3**	84.0	**16.0**
2007	234527	**46.9**	84.3	**15.7**
2012	241175	**47.3**	84.3	**15.7**
2009	251744	**48.2**	84.0	**16.0**
2010	249735	**47.8**	83.6	**16.5**
2011	246885	**47.9**	83.3	**16.7**
2012	237735	**47.6**	83.4	**16.6**
